# Endoscopic ultrasound-guided creation of a drainage route combined with third-space endoscopy for esophageal intramural abscess debridement

**DOI:** 10.1055/a-2792-9848

**Published:** 2026-03-02

**Authors:** Tzong-Hsi Lee, Guan-De Li, Chao-Yu Liu, Chung-Tsui Huang, Chi-Chu Lo, Chen-Shuan Chung

**Affiliations:** 1 46608 Division of Gastroenterology and Hepatology, Department of Internal Medicine, Far Eastern Memorial Hospital, New Taipei City, Taiwan; 2 46608 Division of Thoracic Surgery, Department of Surgery, Far Eastern Memorial Hospital, New Taipei City, Taiwan; 3 Taiwan Association for the Study of Intestinal Diseases (TASID), Taoyuan City, Taiwan; 4 46608 Program A, Department of Electrical Engineering, Yuan Ze University, Taoyuan, Taiwan

**Fig. 1:**
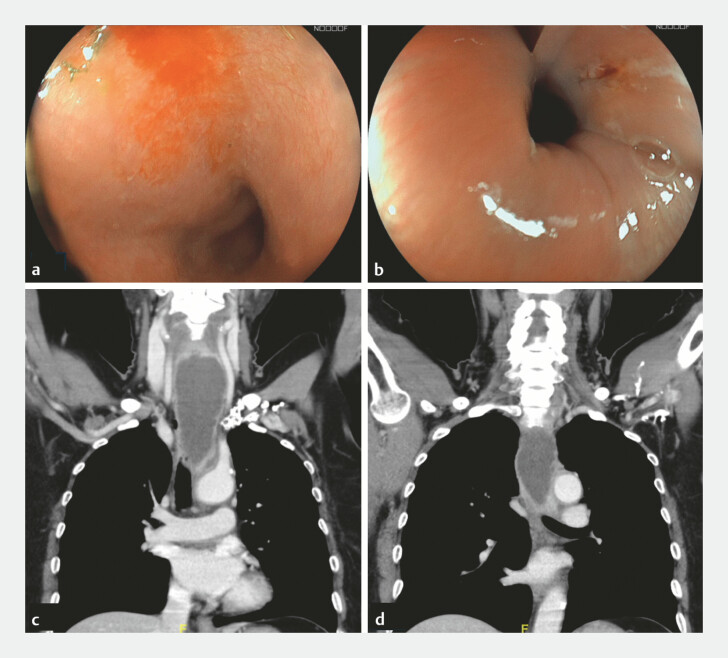
Evolution of esophageal mucosal erosions into an esophageal intramural abscess. **a, b** Multiple erosions were noted at the esophageal inlet and mid-esophagus during the initial EGD. **c, d** Contrast-enhanced CT revealed a large (12.8 cm × 4.5 cm × 3.2 cm) fluid-filled collection with marginal enhancement along the para-esophageal region at the C5–T5 spine level, suggestive of a para-esophageal abscess. CT, computed tomography; EGD, esophagogastroduodenoscopy.

A 62-year-old woman presented with odynophagia after accidental fish bone ingestion. Subsequent esophagogastroduodenoscopy (EGD) identified a 2-cm fish bone lodged at the upper esophageal inlet, which was removed with retrieval forceps. Multiple mucosal erosions at the esophageal inlet and mid-esophagus were noted ( Fig. 1 **a, b** ). Two weeks later, she developed persistent odynophagia, dysphagia and fever, and laboratory tests showed leukocytosis. Computed tomography (CT) revealed an esophageal para-esophageal abscess extending from the upper to mid-esophagus ( Fig. 1 **c, d** ).

Endoscopic ultrasound (EUS) confirmed an abscess located in the submucosal space. EUS-guided transmural drainage was performed using a 19-gauge needle (Expect Slimline Needle, Boston Scientific, Marlborough, MA) and guidewire-assisted tract creation, followed by the placement of a 10-Fr double-pigtail stent (Advanix Biliary Stents, Boston Scientific, Marlborough, MA) for continuous drainage. A mucosal incision was then made to access the submucosal tunnel, allowing endoscopic entry into the abscess cavity for debridement ( Video 1 ). A nasogastric tube was inserted for postoperative feeding.

EUS-guided creation of a drainage route combined with third-space endoscopy for esophageal intramural abscess debridement. EUS, Endoscopic ultrasound.Video 1

**Fig. 2:**
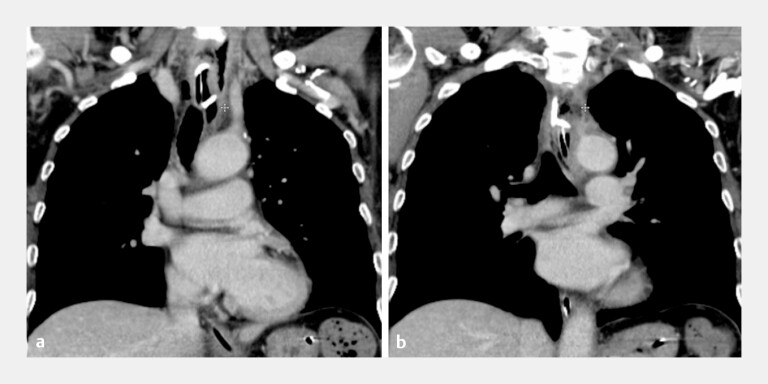
Resolution of the esophageal intramural abscess after EUS-guided double-pigtail stent placement combined with submucosal endoscopic debridement. **a, b** A CT scan performed 3 days later showed the complete resolution of the esophageal abscess. CT, computed tomography; EUS, endoscopic ultrasound.

Symptoms improved markedly after the procedure, and cultures obtained from the abscess cavity grew *Streptococcus anginosus* , *Streptococcus constellatus* and *Eikenella corrodens* , consistent with oral flora. Follow-up CT and EGD 3 days later demonstrated the complete resolution of the abscess ( Fig. 2 **a, b** ). The stent was removed 1 week later, and the mucosal entry site was closed with endoclips (MANTIS Clip, Boston Scientific, Marlborough, MA and SureClip, Micro-Tech Endoscopy, USA; Video 1 ). The patient resumed normal oral intake without further complications.

Submucosal esophageal abscess is a rare condition that may arise from mucosal injury without full-thickness perforation. EUS can delineate both the depth and the layer of involvement with greater accuracy as demonstrated in this case. While antibiotics are typically first-line therapy, extensive abscesses may require intervention 1 . This case highlights a novel, minimally invasive approach combining endoscopic ultrasound-guided drainage and third-space endoscopic debridement, achieving rapid recovery without surgical intervention.

Endoscopy_UCTN_Code_CCL_1AB_2AC_3AZ
